# A Comparison of Patient and Provider Perspectives on an Electronic Health Record–Based Discharge Communication Tool: Survey Study

**DOI:** 10.2196/60506

**Published:** 2025-01-29

**Authors:** Dorothy Yingxuan Wang, Eliza Lai-Yi Wong, Annie Wai-Ling Cheung, Kam-Shing Tang, Eng-Kiong Yeoh

**Affiliations:** 1 JC School of Public Health and Primary Care, The Chinese University of Hong Kong, Hong Kong, China (Hong Kong); 2 Centre for Health Systems & Policy Research, JC School of Public Health and Primary Care, The Chinese University of Hong Kong, Hong Kong, China (Hong Kong); 3 Kwong Wah Hospital, Hospital Authority, Hong Kong, China (Hong Kong)

**Keywords:** older adult, gerontology, geriatric, old, older, elderly, aging, aged, post-acute care, communication, satisfaction, medication information, patient-provider comparison, technology-based intervention, technology acceptance model, discharge, EHR, record, portal, cross-sectional, survey, questionnaire, experience, attitude, opinion, perception, perspective, acceptance, adoption, design, user experience

## Abstract

**Background:**

Hospital discharge for older adult patients carries risks. Effective patient-provider communication is crucial for postacute care. Technology-based communication tools are promising in improving patient experience and outcomes. However, there is limited evidence comparing patient and provider user experiences on a large-scale basis, hindering the exploration of true patient-provider shared understanding.

**Objective:**

This study aimed to evaluate an electronic health record–based discharge communication tool by examining and comparing patient and provider perspectives.

**Methods:**

This study comprised a cross-sectional self-administered staff survey and a pre-post cross-sectional patient survey. Physicians, nurses, and older adult patients aged 65 years and older discharged from 4 public hospitals were included. Patient-provider comparison items focused on 3 aspects of the design quality of the tool (information clarity, adequacy, and usefulness) and overall satisfaction with the tool. In addition, patients’ experience of discharge information and their medication-taking behaviors before and after the program implementation were compared based on a validated local patient experience survey instrument. Providers’ perceived usefulness of this tool to their work and implementation intentions were measured based on the technology acceptance model to enhance understanding of their experiences by conducting structural equation modeling analysis.

**Results:**

A total of 1375 and 2353 valid responses were received from providers and patients, respectively. Patients’ overall satisfaction with this communication tool is significantly higher than providers’, and patients rated the information clarity and usefulness presented by this tool higher as well (*P*<.001). However, patients rated information adequacy significantly lower than providers (*P*<.001). Meanwhile, patients reported a significant improvement in their experience of discharge medication information, and fewer patients reported side effects encounters after the program implementation (126/1083, 11.6% vs 111/1235, 9%; *P*=.04). However, providers showed inconsistent implementation fidelity. Providers’ perceived quality of the tool design (β coefficient=0.24, 95% CI 0.08-0.40) and perceived usefulness to their work (β coefficient=0.57, 95% CI 0.43-0.71) significantly impacted their satisfaction. Satisfaction can significantly impact implementation intentions (β coefficient=0.40, 95% CI 0.17-0.64), which further impacts implementation behaviors (β coefficient=0.16, 95% CI 0.10-0.23).

**Conclusions:**

A notable disparity exists between patients and health care providers. This may hinder the achievement of the tool’s benefits. Future research should aim for a comprehensive overview of implementation barriers and corresponding strategies to enhance staff performance and facilitate patient-provider shared understanding.

## Introduction

At hospital discharge, health care providers play a crucial role in delivering comprehensive medication information, including side effects and warnings, to ensure safe medication therapy during patients’ postacute care [1]. However, previous literature has reported that older adult patients often lack awareness or understanding of their medication regimen after being discharged home [2-4]. Insufficient knowledge is associated with their suboptimal adherence to treatment [5,6], an elevated likelihood of adverse events [7], increased readmissions and emergency department visits [8], and burden on the health care system [9].

A wide array of communication strategies has been documented in the literature with the aim of facilitating information provision by health care providers and enhancing patient awareness and understanding of health-related information [10]. Their effectiveness in reducing readmissions and enhancing patient satisfaction was supported by a recent meta-analysis [1]. Notably, information technology–based communication practices have emerged as a prominent and preferred mode for delivering discharge information, as highlighted in literature reviews [11]. In addition, a systematic review concluded that computer-enabled discharge communication interventions improve both patient and provider satisfaction and reduce perceived adverse events [12]. A Cochrane review further indicated that computer-generated reminders presented on paper can enhance the quality of care [13]. However, there is a scarcity of research comparing the perspectives of older adult patients and health care providers with concordance measures for a large-scale technology-based discharge communication tool. Measuring and comparing the alignment between patient and provider perspectives enables the unveiling of true shared understanding in terms of discharge education [14].

In Hong Kong, the provision of discharge medication information, particularly regarding side effects and warnings, was found to be suboptimal, according to a regular patient experience survey [15]. In 2017, the Hospital Authority developed a computer-generated written medication reminder called the postdischarge information summary (PDIS) to address this issue [16]. The key components of the PDIS were co-designed by a multidisciplinary program team consisting of government officials, clinicians, quality and safety representatives, and technology experts. The first component includes a salient medication reminder, a computer-based drug database encompassing 58 prescribed medications for local older adult patients, and 235 most pertinent side effects and warning items. This database underwent validation through 3 rounds of Delphi expert consensus meetings [17]. The second component comprises a list of follow-up appointments across all Hong Kong public hospitals. The PDIS system generates personalized information by integrating into the electronic health record (EHR). During discharge, physicians or nurses are required to print the written summary through the PDIS system and distribute it to discharged patients or their caregivers, along with a detailed explanation of its contents. No teach-back was required at the moment of program introduction. A comparison of the discharge communication workflow between usual practice and PDIS-incorporated practice is shown in Multimedia Appendix 1.

The objective of this study is to evaluate this EHR-based discharge communication tool by examining and contrasting the perspectives of both older adult patients and health care providers.

## Methods

### Study Design

This study comprises a self-administered cross-sectional staff survey and a pre-post cross-sectional patient survey. The pre-post patient surveys were conducted among 2 different patient groups. The STROBE (Strengthening the Reporting of Observational Studies in Epidemiology) guideline [18] was used to strengthen the reporting process (Checklist 1).

### Setting and Sampling

The study involved 4 piloting public tertiary hospitals representing 3 out of 7 geographical clusters of Hong Kong. The PDIS was introduced in a phased manner within the geriatric and medicine department in January 2018 [16]. For the staff survey, all physicians and nurses involved in the PDIS implementation were invited to participate in the study. The surveys were conducted at least 6 months after the PDIS implementation, which spanned from August 2018 to June 2019. Paper-based promotion leaflets, invitation letters, and questionnaires were distributed through designated coordinators in each hospital. This survey was conducted anonymously and on a voluntary basis.

For the patient survey, the sample size was determined based on the inpatient discharge statistics for patients aged 65 years or older in 2015, as provided by the Hospital Authority. In order to achieve a precision level of ±4% with a 95% CI, a minimum of 1450 respondents was required for pre-post rounds. Assuming a 50% response rate, at least 2900 patients were randomly selected from the discharge records for each round. Responses from caregivers acting as surrogates were accepted if patients were unable to respond independently. Readmitted cases and day patients were excluded. Within 14 days of their discharge, patients were contacted by telephone. The pre-post survey was conducted from June to December 2017 and May to December 2018, accordingly.

The staff survey used English and the patient version used Chinese. We used English in staff survey because English is their working language and they are proficient in English. Furthermore, staff surveys are typically conducted in English in Hong Kong, so it was assumed that participants would feel comfortable with this language. To ensure language did not pose a barrier to participation or affect the responses collected, we decided to use Chinese for the patient survey. We do not anticipate any language concordance issues because the researchers who were responsible for collecting patient responses were well-trained before the survey to ensure they can correctly convey the meaning of the questions.

### Theoretical Framework

The staff survey collected information on providers’ practicing behaviors and user experience, adapted by the technology acceptance model (TAM) [19]. TAM has been designed to investigate why individuals adopt a specific technology and has been widely used in different settings [20].

According to the TAM, the perceived usefulness of the technology can impact users’ behavior intention, which can be a determinant of users’ actual behaviors. We added another domain named design quality to capture providers’ perceived information quality of the tool. We also measured the overall satisfaction of the PDIS experience. Existing literature suggests that design quality can significantly influence staff adoption and implementation of the technologies [21]. The traditional TAM component of “Perceived Ease of Use” was not fully applicable in this context, as our primary aim was to compare the perceptions of patients and staff regarding their user experiences and perceptions of the PDIS. However, “Perceived Ease of Use” tends to be more relevant to staff, given that staff interact with the information system while patients interact with a paper-based version rather than a technology. Consequently, we adopted “Design Quality” as a domain to facilitate a meaningful comparison of perspectives between these 2 groups. In addition, we sought to streamline our survey to enhance response rates, particularly among staff who may have limited time to participate. Thus, we decided to use design quality as a domain in place of perceived ease of use.

**Figure 1.**
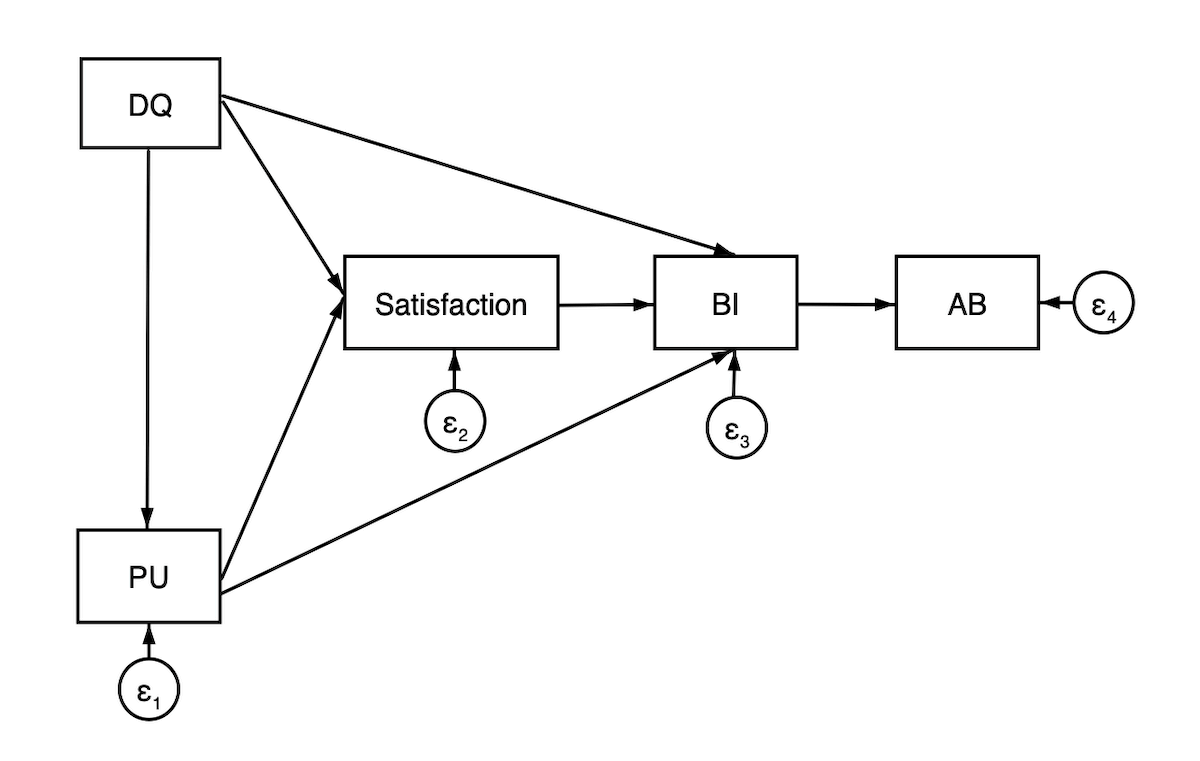
Conceptual framework of the factors impacting implementation fidelity. AB: actual behavior; BI: behavior intention; DQ: design quality; PU: perceived usefulness; “ε1”, “ε2”, “ε3”, and “ε4” were the residual errors.

We hypothesized that (1) design quality would have an impact on perceived usefulness, satisfaction, and behavior intention; (2) perceived usefulness would impact satisfaction and behavior intention; (3) satisfaction would impact behavior intention; and (4) behavior intention would impact actual behavior (Figure 1).

### Measurements

#### The Staff Survey

##### Perceived Usefulness

In total, 4 items were used to measure perceived usefulness: (1) “PDIS supports my medication education,” (2) “PDIS enhances my communication with patients or caregivers,” (3) “PDIS enhances my job efficiency,” and (4) “I find PDIS useful in my job.” The Cronbach α for this domain was 0.97, indicating the good reliability. A Likert scale ranging from 0 (“strongly disagree”) to 10 (“strongly agree”) was used for these 4 items.

##### Design Quality

Design quality was measured using 3 items developed by the research team: (1) “The information provided by the PDIS is clear,” (2) “The information provided by the PDIS is sufficient,” and (3) “The information provided by the PDIS is useful to your patients or caregivers.” The Cronbach α for this domain was 0.92, indicating good reliability. A 10-point Likert scale was used to measure these three items ranging from 0 (“strongly disagree”) to 10 (“strongly agree”).

##### Behavior Intention

One item, which was investigator team constructed, was adopted to measure behavior intention: “I am willing to use PDIS to deliver patient discharge information in the future.” The 10-point Likert scale was used (0: strongly disagree to 10: strongly agree) for this item.

##### Satisfaction

Satisfaction was measured using a single item: “How would you rate your experience of delivering patient discharge information using PDIS?” A 10-point Likert scale ranging from 0 (“very poor”) to 10 (“very good”) was used for this question.

##### Actual Behaviors

The actual behavior was defined as the implementation fidelity and measured by the frequency of distributing, reading, and explaining the PDIS to patients and caregivers with a 3-level ordinal scale (never, sometimes, and always). A composite score for actual behavior was created by converting the ordinal scale for each behavior into to a 0, 1, and 2 scale and summing it up across the 3 practicing behaviors (distribution, read, and explanation) to generate a total score.

##### Qualitative Comments

Free-text fields were provided to solicit provides’ comments on their PDIS experiences. Respondents were prompted with the following question: “Is there anything you would like to tell us about the PDIS (eg, things that were particularly good, areas for improvement, or any other comments)?”

##### Demographic Information

Demographic information was collected in the final section of the survey. For example, we asked their self-reported gender, age, working experience in years, and professional role (eg, interns, residents, specialists, associate consultants, and consultants for physicians; enrolled nurses, registered nurses, advanced practice nurses, and ward managers for nurses).

### The Patient Survey

The pre-post patient surveys consist of the following sections.

#### The Patient Experience

The items were drawn from the validated local assessment tool, the Short-form Hong Kong Inpatient Experience Questionnaire (SF-HKIEQ) [22], soliciting patient agreement on the clarity, adequacy, and usefulness of discharge medication information, including side effects and warnings, using an ordinal scale (yes, to some extent, or no) or Likert scale of 0‐10 from strongly disagree to strongly agree; the Cronbach α is 0.87, indicating good reliability. The overall satisfaction of the PDIS experience was also solicited. A 0‐10 Likert scale of strongly disagree/very bad to strongly agree/very good was applied to measure the items. In the patient survey, consistent with the previous analysis approach for the SF-HKIEQ, questions with an ordinal scale were converted to a 0, 5, and 10 scale and aggregated to calculate the mean and SD.

#### Self-Reported Medication-Taking Behavior

This section was developed based on the 4-item Morisky, Green, Levine (MGL) scale [23] and relevant studies [24]. Furthermore, 1 item was adopted from the MGL scale: “Ever forget to take medicines?” In addition, 2 items were investigator-constructed: “Whether you were compliant with their medication regimen” and “Whether you have ever experienced medication side effects.” These 3 items were measured by binary responses (yes or no).

#### Design Quality

The patient postsurvey includes the same question of the design quality domain of the staff survey, allowing comparative analysis: (1) “The PDIS information is clear to me or patients,” (2) “The PDIS information is enough to me or patients,” and (3) “The PDIS information is useful to self-care or care for patients.” A 10-point Likert scale was used (0: “strongly disagree” to 10: “strongly agree”). The Cronbach α was 0.94, indicating a high reliability.

#### Free-Text Field

A free-text section was added to solicit patients’ comments on the PDIS experiences. Similarly with staff survey, we asked patients “what information do you need that was not provided by the PDIS, and any other comments?”

#### Patient Characteristics

Patient characteristics, such as their age, self-reported gender, education level (primary, secondary, college, and above), whether received the government subsidy, the comprehensive social security assistance designed for people whose income is not sufficient to meet basic needs), living arrangements (living alone or living with others), chronic conditions (including heart disease, hypertension, type 2 diabetes, and cancer), and self-reported quality of life using visual analog scale of the EQ-5D-5L Hong Kong [25], were also asked.

### Data Analysis

The statistical analysis was performed utilizing R version 4.0.5 (R Project for Statistical Computing) and Stata version 18 for Mac (StataCorp). Provider and patient demographic information were summarized as means and percentages using descriptive statistical analysis. The staff survey and patient survey were analyzed separately. For the staff survey, subgroup analysis was conducted to evaluate the differences among physicians and nurses. The practicing behavior frequency and PDIS experiences were compared using the Pearson chi-square test and Mann-Whitney *U* test. The Mann-Whitney *U* test was used due to the outcome variables were skewed. In order to understand the relationship between the determinants of the behaviors like design quality, perceived usefulness, behavior intention, satisfaction of the PDIS, and the actual behavior, as well as the relationship between different determinants, the covariance-based structural equation modeling (CB-SEM) was applied. The goodness of fit index, comparative fit index (CFI), root mean square error approximation (RMSEA), Tucker-Lewis index (TLI), and standard root mean squared residual (SRMSR) were used to evaluate the goodness-of-fit of the model. The direct effects, indirect effects, and total effects between the core variables were assessed using a bootstrapping approach (n=2000). Standardized path coefficients (β) were used to estimate the path relationships. Statistical significance was set at *P*=.05.

For the patient survey, changes in patient experience regarding medication information before and after PDIS implementation were compared using the Mann-Whitney *U* test due to the outcome variables were skewed. The difference between self-reported side effects encounters and compliance was assessed using the Pearson chi-square test.

To identify the disparity between staff and patient. the shared questions related to PDIS experience (eg, information clarity, adequacy, and usefulness, and the overall experience of the PDIS) in the staff and patient postsurveys were compared using the Mann-Whitney *U* test. For the qualitative comments from both staff and patient, thematic synthesis [26] was applied to identify and compare the common themes in free-text comments for the PDIS program between patients and providers. Furthermore, 2 coders independently coded all the utterances of patients and staffs to generate the initial codes to achieve consensus on coding. The disagreement were formally resolved through several discussions with a senior researcher experienced in qualitative study. After that, the inductive analysis was performed by one coder to generalize initial codes into overarching themes. The initial summary themes were developed and discussed with the senior research to reach consensus. The final list of themes was reviewed and consented by the research team. The theme list were identical for patient and staff and the frequency of each theme were counted in order to compare the pattern for patients and staffs.

### Ethical Considerations

Ethical approval has been provided by the Joint Chinese University of Hong Kong – New Territories East Cluster Clinical Research Ethics Committee in compliance with the Declaration of Helsinki (CREC 2019.436). For the staff survey, implied consent was applied as participating staff members returned the completed questionnaires to the research team. For the patient survey, verbal consent was obtained from patients over the phone before the survey. All data were deidentified to protect participants’ privacy and confidentiality.

## Results

### Comparative Analysis

A total of 1375 providers completed the survey with a 76% response rate, comprising 966 (72%) female participants, 650 (50%) participants aged 18‐29 years old, 595 (50%) participants with 0‐5 years of working experience, and 1216 (88%) nurses (Table 1).

**Table 1.** Demographic information of health care providers.

Characteristics		Total (N=1375)	Doctors (n=159)	Nurses (n=1216)
**Sex, n (%)** ^a^				
	Female	966 (72)	55 (35)	911 (77)
	Male	382 (28)	103 (65)	279 (23)
**Age (years), n (%)** ^b^				
	18-29	650 (50)	52 (34)	598 (52)
	30-39	349 (27)	50 (32)	299 (26)
	40-49	206 (16)	32 (21)	174 (15)
	50-59	86 (6.6)	21 (14)	65 (5.7)
	>59	5 (0.4)	0 (0)	5 (0.4)
**Working experience (years), n (%)** ^c^				
	0-5	595 (50)	46 (34)	549 (53)
	6-10	278 (24)	25 (18)	253 (24)
	11-15	94 (8.0)	23 (17)	71 (6.8)
	16-20	125 (11)	18 (13)	107 (10)
	20-25	42 (3.6)	13 (9.6)	29 (2.8)
	26-30	37 (3.1)	10 (7.4)	27 (2.6)
	>30	9 (0.8)	1 (0.7)	8 (0.8)

a 31 participants excluded from analysis due to missing information.

b 82 participants excluded from analysis due to missing information.

c 185 participants excluded from analysis due to missing information.

From the patient side, we received 2353 valid responses, including 1109 (47%) and 1244 (53%) responses collected through the pre- and postsurveys, respectively. The response rate was 55.5% for the presurvey and 59.4% for the postsurvey. The demographic composition was similar between the pre- and postsurvey groups, except that 6.4% (presurvey group: 853/1106; postsurvey group: 1023/1226) more participants were receiving the government subsidy in the postsurvey group (*P*<.001) (Table 2).

**Table 2.** Demographic information of older adult patients.

Characteristics		Total (N=2353)	Presurvey (n=1109)	Postsurvey (n=1244)
Age (years), mean (SD)		77.48 (7.98)	77.54 (8.00)	77.65 (7.93)
**Sex, n (%)**				
	Female	1070 (45.5)	517 (46.6)	553 (44.5)
	Male	1283 (54.5)	592 (53.4)	691 (55.5)
**Education, n (%)** ^a^				
	≤Primary	1461 (62.8)	695 (63.4)	946 (62.3)
	Secondary	702 (30.2)	341 (31.1)	361 (29.4)
	≥College	162 (7)	60 (5.5)	102 (8.3)
**Living status, n (%)** ^b^				
	Living alone	292 (12.4)	138 (12.4)	154 (12.4)
	Living with others	2056 (87.6)	971 (87.6)	1085 (87.6)
**Government subsidy, n (%)** ^c^				
	Yes	1880 (80.5)	853 (77.1)	1023 (83.5)
	No	456 (19.5)	253 (22.9)	203 (16.5)
**Heart diseases, n (%)** ^d^				
	Yes	844 (36.1)	398 (35.9)	446 (36.3)
	No	1492 (63.9)	710 (64.1)	782 (63.7)
**Hypertension, n (%)** ^e^				
	Yes	1380 (59.1)	671 (60.6)	709 (57.7)
	No	956 (40.9)	437 (39.4)	519 (42.3)
**Type 2 diabetes, n (%)** ^f^				
	Yes	754 (32.3)	358 (32.3)	396 (32.2)
	No	1582 (67.7)	750 (67.7)	832 (67.8)
**Cancer, n (%)** ^g^				
	Yes	156 (6.7)	85 (7.7)	71 (5.8)
	No	2181 (93.3)	1023 (92.3)	1158 (94.2)
**Length of stay (day), n (%)** ^h^				
	0-3	1256 (53.7)	592 (53.6)	664 (53.7)
	4-7	639 (27.3)	296 (26.8)	343 (27.7)
	>7	446 (19.1)	216 (19.6)	230 (18.6)
EQ-5D-VAS, mean (SD)^i^		66.61 (18.51)	68.37 (17.39)	65.04 (19.33)
**Discharge day, n (%)**				
	Weekday	1916 (81.5)	909 (82)	1007 (80.9)
	Weekend	437 (18.5)	200 (18)	237 (19.1)

a 29 participants excluded from analysis due to missing information.

b 5 participants excluded from analysis due to missing information.

c 17 participants excluded from analysis due to missing information.

d 17 participants excluded from analysis due to missing information.

e 17 participants excluded from analysis due to missing information.

f 17 participants excluded from analysis due to missing information.

g 16 participants excluded from analysis due to missing information.

h 12 participants excluded from analysis due to missing information.

i 109 participants excluded from analysis due to missing information.

**Figure 2.**
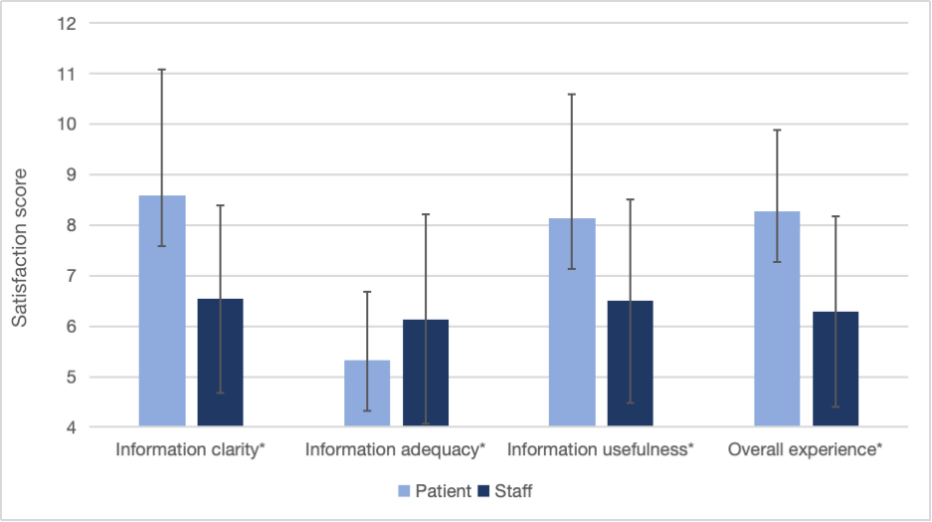
Comparison of patient and health care providers’ postdischarge information summary experiences. *P* value was obtained from the Mann-Whitney *U* test. *P<.001.

The comparative evaluation showed that patients consistently provided significantly higher ratings for their overall PDIS satisfaction compared with providers (mean 8.28, SD 1.60 vs mean 6.29, SD 1.88, respectively; *P*<.001) (Figure 2). Specifically, patients reported higher ratings for information clarity (mean 8.58, SD 2.50 vs mean 6.54, SD 1.86, respectively; *P*<.001) and usefulness of the PDIS (mean 8.14, SD 2.46 vs mean 6.50, SD 2.02, respectively; *P*<.001). On the contrary, patients were inclined to receive more information through the PDIS (mean 5.33, SD 1.35 vs mean 6.14, SD 2.08, respectively; *P*<.001).

**Figure 3.**
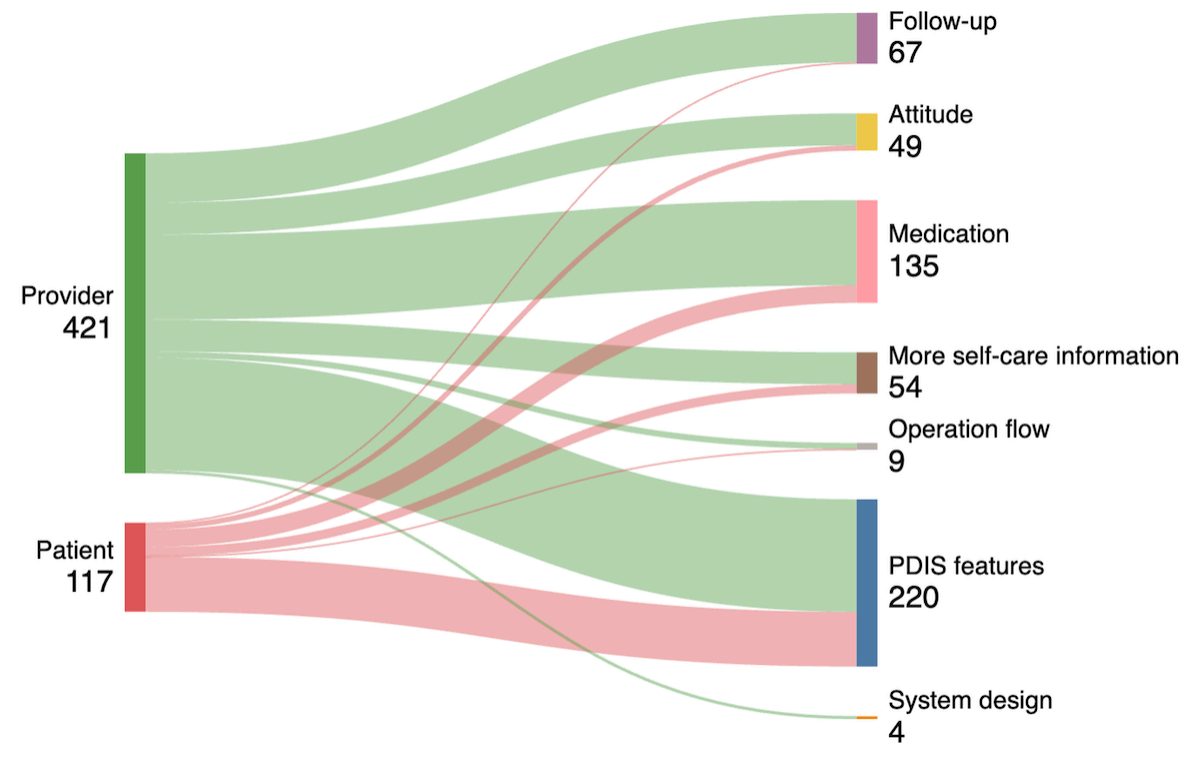
Comparison of the qualitative comments on the postdischarge information summary between patients and health care providers. PDIS: postdischarge information summary.

Figure 3 displays the similarities and differences between patients and providers regarding their comments on PDIS experiences. A total of 538 comments were received (421 were from providers and 117 were from patients). The most frequently commented aspect was PDIS features for both providers (147/421, 35%) and patients (73/117, 62%). However, the specific area of the feature was different. Providers emphasized the need for broader coverage of the drug databases (60/147, 41%) and the lack of multiple language versions (59/147, 40%), while patients’ concerns revolve around the inconvenience of medication names and follow-up information being in English (45/73, 61%), the discomfort with medical jargon (9/73, 13%), and font size (7/73, 10%). In addition, providers frequently commented on the content of the PDIS form. For example, 27% (114/421) of the comments were related to the medication listed on the PDIS and emphasized the need for additional medication details such as medication changes, indications, and instructions.

### Staff Survey

Analysis of the practicing behaviors (Table 3) revealed that 72.1% (n=922) of the providers reported being able to consistently distribute the PDIS form to patients or caregivers, whereas 56% (n=667) stated they could consistently explain its content. Subgroup analysis demonstrated significant variations across different roles. Regarding distribution, 78% (n=915) of nurses reported always doing so, compared with 6.4% (n=7) of doctors (*P*<.001). Similarly, 57% (n=666) of nurses reported always explaining the content, compared with 4% (n=1) of doctors (*P*<.001).

**Table 3.** Health care providers’ practicing behaviors and experience of the postdischarge information summary.

Items			Total (N=1375)	Doctors (n=159)	Nurses (n=1216)	*P* value^a^
**PDIS^b^ implementation behavior, n (%)**						
	**Distribute**		1234 (89.7)	109 (68.6)	1170 (96.2)	<.001
		Always	922 (72)	7 (6.4)	915 (78)	
		Sometimes	261 (20)	17 (16)	244 (21)	
		Never	96 (7.5)	85 (78)	11 (0.9)	
	**Read**		1190 (86.5)	26 (16.4)	1154 (94.9)	<.001
		Always	730 (61)	5 (19)	725 (62)	
		Sometimes	425 (36)	16 (62)	409 (35)	
		Never	35 (2.9)	5 (19)	30 (2.6)	
	**Explain**		1190 (86.5)	26 (16.4)	1164 (95.7)	<.001
		Always	667 (56)	1 (3.8)	666 (57)	
		Sometimes	475 (40)	11 (42)	464 (40)	
		Never	48 (4)	14 (54)	34 (2.9)	
**Perceptions of PDIS experiences, mean (SD)**						
	**Perceived design quality**					
		PDIS information is clear to your patients or careers	6.54 (1.86)	6.50 (1.61)	6.54 (1.86)	.67
		PDIS information is adequate to your patients or careers	6.14 (2.07)	6.24 (1.61)	6.14 (2.08)	.93
		PDIS information is useful for patients or careers	6.50 (2.02)	6.69 (1.81)	6.50 (2.02)	.70
	**Perceived usefulness**					
		PDIS supports my medication education to patients or careers	6.35 (2.07)	6.08 (1.81)	6.36 (2.07)	.32
		Patient-provider communication becomes more effective with PDIS	6.21 (2.05)	6.00 (1.55)	6.21 (2.06)	.34
		PDIS enhances my job efficiency	5.97 (2.20)	6.08 (1.72)	5.97 (2.21)	.83
		PDIS is useful in my job	6.03 (2.16)	6.12 (1.68)	6.02 (2.17)	.97
	**Behavior intention**					
		I am willing to use PDIS	6.09 (2.18)	6.12 (1.63)	6.09 (2.19)	.72
	**Overall satisfaction**					
		Overall rating of PDIS user experiences	6.29 (1.88)	6.56 (1.51)	6.28 (1.88)	.75

a *P* value was obtained from the chi-square test and Mann-Whitney *U* test.

b PDIS: postdischarge information summary.

**Figure 4.**
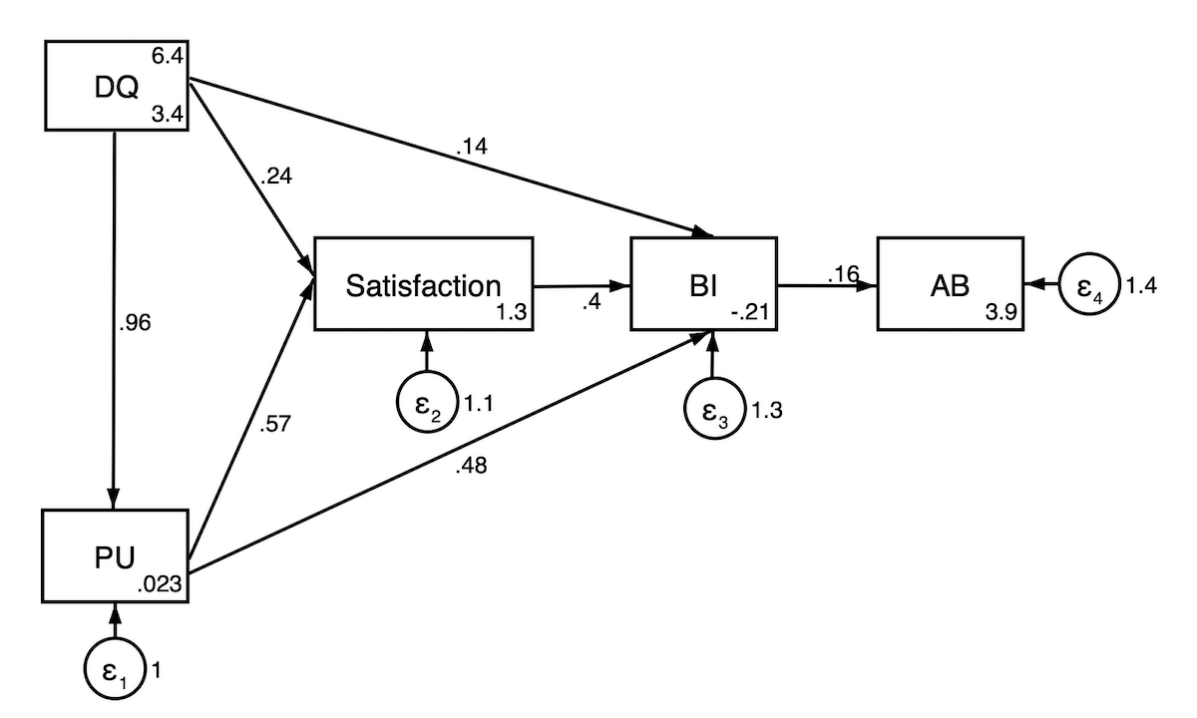
Structural equation modeling for factors impacting the providers’ implementation fidelity. AB: actual behavior; BI: behavior intention; DQ: design quality; PU: perceived usefulness; “ε1”, “ε2”, “ε3”, and “ε4” were the residual errors.

Physicians and nurses indicated moderate satisfaction with the design quality and perceived usefulness of the PDIS to their work, as reflected by mean agreement scores ranging between 5.96 and 6.54 (Table 3). The subgroup analysis did not identify any differences between professional roles regarding user experiences. The CB-SEM analysis (Figure 4) showed that design quality significantly impacted their perceived usefulness (β coefficient=0.96, 95% CI 0.90-1.01) and behavior intention (β coefficient=0.14, 95% CI 0.06-0.21). In addition, perceived usefulness had a significant impact on behavior intention (β coefficient=0.48, 95% CI 0.26-0.70). Furthermore, behavior intention had a significant impact on the actual behavior (β coefficient=0.16, 95% CI 0.10-0.23). In addition, satisfaction can be significantly impacted by the design quality (β coefficient=0.24, 95% CI 0.08-0.40) and perceived usefulness (β coefficient=0.57, 95% CI 0.43-0.71). The structural equation modeling (SEM) model presents a good fit overall, with all indicators exceeding the recommended thresholds (Table 4). The results of the indirect effects and total effects can be found in MultimediaAppendices 2  3. In total, 3 mediating pathways were identified: (1) an indirect pathway from design quality through satisfaction to behavior intention (β coefficient=0.770, 95% CI 0.700‐0.841, proportion mediated=15.1%); (2) an indirect pathway from perceived usefulness through satisfaction to behavior intention (β coefficient=0.228, 95% CI 0.185‐0.272, proportion mediated=32.5%); and (3) an indirect pathway from design quality through perceived usefulness to satisfaction (β coefficient=0.544, 95% CI 0.482‐0.605, proportion mediated=30.8%).

**Table 4.** Direct effects for the model^a^.

Relationship	Standardized estimates (95% CI)		Remarks
Design quality → Perceived usefulness	0.955 (0.923-0.987)		Supported
Design quality → Satisfaction	0.242 (0.151‐0.333)		Supported
Perceived usefulness → Satisfaction	0.569 (0.487-0.651)		Supported
Design quality → Behavior intention	0.137 (0.031-0.242)		Supported
Perceived usefulness → Behavior intention	0.476 (0.371-0.582)		Supported
Satisfaction → Behavior intention	0.402 (0.288-0.515)		Supported
Behavior intention → Actual behavior	0.164 (0.131-0.197)		Supported

a Model fit: *χ*_441_=3088.344, *P*<.001; root mean square error approximation=0.052; comparative fit index=0.997; Tucker-Lewis index=0.990; standard root mean squared residual=0.021.

### Patient Survey

Table 5 showed significant improvements in patient experience related to overall discharge information clarity (mean 8.18, SD 1.69 vs mean 7.93, SD 1.84, respectively; *P*=.002), adequacy (mean 8.15, SD 1.76 vs mean 7.92, SD 1.93, respectively; *P*=.01), and usefulness (mean 8.26, SD 1.70 vs mean 8.06, SD 1.80, respectively; *P*=.02). In addition, a significant increase was found in information adequacy for both side effects (mean 9.6, SD 2.0 vs mean 8.6, SD 3.4, respectively; *P*<.001) and warnings (mean 9.7, SD 1.8 vs mean 9.2, SD 2.7, respectively; *P*=.004). Only warning information reached statistically significant improvement in clarity (mean 8.77, SD 2.32 vs mean 8.45, SD 2.45, respectively; *P*=.03) and usefulness (mean 8.7, SD 2.45 vs mean 8.44, SD 2.39, respectively; *P*=.03).

**Table 5.** Older adult patients’ or caregivers’ perceptions of discharge medication information and medication-taking behavior between pre- and postsurvey groups.

Items			Total (N=2353)	Presurvey (n=1109)	Postsurvey (n=1244)	*P* value^a^
**Perspectives of the discharge medication information**						
	**Clarity, mean (SD)**					
		Side effects	8.47 (2.50)	8.31 (2.63)	8.60 (2.39)	.10
		Warning signs	8.61 (2.39)	8.45 (2.45)	8.77 (2.32)	.03
		Overall	8.06 (1.77)	7.93 (1.84)	8.18 (1.69)	.003
	**Adequacy, mean (SD)**					
		Side effects	9.2 (2.8)	8.6 (3.4)	9.6 (2.0)	<.001
		Warning signs	9.4 (2.3)	9.2 (2.7)	9.7 (1.8)	.004
		Overall	8.04 (1.85)	7.92 (1.93)	8.15 (1.76)	.02
	**Usefulness, mean (SD)**					
		Side effects	8.47 (2.46)	8.34 (2.51)	8.57 (2.42)	.12
		Warning signs	8.57 (2.42)	8.44 (2.39)	8.70 (2.45)	.03
		Overall	8.17 (1.75)	8.06 (1.80)	8.26 (1.70)	.01
**Medication-taking behaviors**						
	**Self-reported side effects encounter, n (%)**		2318 (86.1)	1083 (85.6)	1235 (86.6)	.04
		Yes	237 (10.2)	126 (11.6)	111 (9)	
		No	2081 (89.8)	957 (88.4)	1124 (91)	
	**Self-reported medication compliance, n (%)**		2325 (86.4)	1085 (85.8)	1240 (87)	.39
		Yes	2226 (95.7)	1043 (96.1)	1183 (95.4)	
		No	99 (4.3)	42 (3.9)	57 (4.6)	

a *P* value was obtained from the Mann-Whitney *U* test and chi-square test.

No statistically significant difference was found between pre- and postsurvey groups in the percentage of self-reported medication compliance. Notably, the postsurvey group had a significantly lower percentage of self-reported side effects encounters (126/1083, 11.6% vs 111/1235, 9%, respectively; *P*=.04). Among participants who reported encountering side effects, the majority (96/111, 86.4%) still followed the medication instructions as prescribed.

## Discussion

### Principal Results

This is the first study to compare the perceptions of older adult patients and health care providers regarding the use of large-scale EHR-based discharge communication tools with concordance measures. There was a noticeable difference in ratings between patients and providers, with patients giving higher ratings in terms of design quality and overall experience of this tool. Qualitative comments indicated that patients and providers have different areas of concern regarding this communication tool. Furthermore, from the health care providers’ side, inconsistent practicing behaviors were found, which were significantly influenced by the implementation intentions (represented as behavioral intentions in the SEM model), overall satisfaction, design quality, and perceived usefulness of the program. However, from the patients’ side, older adults who received the written summary reported improved experiences with discharge information, including information clarity, adequacy, and usefulness.

### Comparison With Previous Work

Providers assigned significantly lower scores to the perceived information clarity of the PDIS to their patients than patients themselves. This difference may be due to the providers’ concern about the challenges associated with older adults’ health literacy [27] and the potential negative consequences of sharing information on side effects, such as patients’ anxiety and non-compliance [28-30]. However, the improved patient-reported ratings of information clarity on medication warnings and overall medication information, significantly fewer side effects encounters, and no evidence of patient noncompliance found by our study and others [31] suggested that taking action is no worse than inaction but yields better outcomes, contrary to the biased perception held by staff members [32]. In order to address staff concerns, facilitate their implementation, and fulfill patients’ needs, rephrasing risk information by using lay language, shorter sentences, supplementing verbal descriptions with visual aids, and presenting medication benefits along with side effects can be considered [33-35].

Providers rated information usefulness for patients or caregivers lower than patients in this study, further impacting their implementation. The lower beliefs on the value of the communication tool for patient care from the provider side may be due to the beliefs that patients may not effectively follow the advice due to a lack of skills or inability to recall, despite clinicians appropriately delivering the instructions [36]. Therefore, it is suggested that using cognitive aid strategies such as teach-back techniques, repetition, demonstration, and reducing the complexity of the information to enhance patients’ capacity to perform self-care tasks and recall of information [10]. The discrepancy can also be attributed to providers’ lack of awareness regarding patient needs, which was also reported by previous studies [37]. Our study results, as well as other research [38], suggest that patients view information about medication side effects and warnings as crucial when making decisions about seeking professional assistance. Patient-provider information gap may not only lead to patient dissatisfaction but also levy stress on providers [37]. Therefore, it is important to leverage patient voices as credible sources and build long-term patient-provider relationships to address this gap.

It is important to note that older adults tend to rely more on health care providers and perceive them as trustworthy sources of information, as reflected in higher satisfaction with received medication information among older adults than younger individuals in a previous study [39]. Therefore, it is crucial to address provider-reported barriers in PDIS implementation to ensure a higher level of program satisfaction and implementation fidelity. Our study found that the design quality and perceived usefulness to providers’ work can hinder their implementation, which is in accordance with previous studies [40,41]. This can be attributed to the low compatibility of the service with providers’ existing workflow [21]. Our study found that providers expressed a preference for enriching the PDIS content with additional medication elements, such as medication changes, indications, and instructions, indicating their priorities for discharge education are not fully met. This suggested that involving frontline implementers as program designers and developers is crucial for program fit, staff self-efficacy, capacity, and commitment [21]. Other than the perceived usefulness and design quality of the program reported in our study, a comprehensive understanding of the complex elements involved in implementation, including context, stakeholders, and organizational factors, is needed [42]. This knowledge facilitates the creation of customized strategies and policies that have a higher likelihood of achieving success [43]. Therefore, conducting implementation research is essential in identifying the barriers and facilitators linked to the implementation of the PDIS to ensure providers’ user experiences are optimized, leading to improved patient access to high-quality care and maintaining a high level of patient experiences among the older adult population.

### Limitations

This study has several limitations. First, self-reported data may be subject to inaccuracies due to social desirability bias. However, including participants from diverse backgrounds may mitigate this limitation to some extent. Second, patient outcomes were not measured in this study, as our focus was on exploring and comparing patient and provider experiences with technology-based communication tools. Given the positive experiences reported by patients, future research could investigate clinical outcomes to further enhance the evidence base. Third, the pre-post survey design for a patient survey without a control group limits our ability to determine whether the observed changes are directly attributable to the PDIS. The cross-sectional design for the staff survey can limit our ability to establish causal inferences regarding factors influencing providers’ inconsistent implementation. Subsequent studies using longitudinal or experimental designs are warranted to understand the causal mechanisms and develop effective strategies to enhance staff performance. Finally, as we did not impose a strict designation of specific professional roles (doctor or nurse) for the tasks involved in PDIS implementation, there may be an underestimation of the effects of the determinants on behavioral intention and actual behaviors.

### Conclusions

EHR-based discharge communication tools have the potential to improve the patient experience with discharge information. However, there is a notable difference in user perceptions between patients and providers. This difference may hinder the full benefits of the program for patients. These findings have implications for future research, particularly in implementation research, where barriers and strategies to enhance staff performance can be investigated. In addition, the study provides valuable insights for organizations seeking to improve patient-provider shared understanding of postacute care plans among older adult patients during hospitalization, particularly through technology-based interventions.

## Supplementary material

10.2196/60506 Multimedia Appendix 1 Pre- and postdischarge information summary discharge communication workflow.

10.2196/60506 Multimedia Appendix 2 Indirect effect for the structural equation modeling model.

10.2196/60506 Multimedia Appendix 3 Total effect of the structural equation modeling model.

10.2196/60506 Checklist 1 STROBE (Strengthening the Reporting of Observational Studies in Epidemiology) statement checklist.

## Abbreviations

CB-SEM: covariance-based structural equation modeling
CFI: comparative fit index
EHR: electronic health record
MGL: 4-item Morisky, Green, Levine
PDIS: postdischarge information summary
RMSEA: root mean square error approximation
SEM: structural equation modeling
SF-HKIEQ: Short-form Hong Kong Inpatient Experience Questionnaire
SRMSR: standard root mean squared residual
STROBE: Strengthening the Reporting of Observational Studies in Epidemiology
TAM: technology acceptance model
TLI: Tucker-Lewis index
