# Supplementary material for: A Comparison of Patient and Provider Perspectives on an Electronic Health Record–Based Discharge Communication Tool: Survey Study
Source: JMIR Aging. 2025 Jan 29;8:e60506. doi: 10.2196/60506 (PMC11796482; doi:10.2196/60506)
Supplement: Multimedia Appendix 1 [file aging-v8-e60506-s001.docx]

**Appendix 1 Pre and Post-PDIS discharge communication workflow**

|  | **Pre-PDIS discharge communication** | **Post-PDIS discharge communication** |
| --- | --- | --- |
| **Information provided** | - A discharge slip (in English) including the diagnosis of the admission, medication and follow-up arrangement. - A QR code on the prescription slip of each medicine for information including brand and generic name, usage, regime, effectiveness, and all possible side effects or interactions. | - A discharge slip (in English) - PDIS (in Chinese) form which includes the most relevant and important warning message and side effects of the prescribed medications and the list of follow-up appointments in public hospitals. - A QR code on the prescription slip of each medicine for information including brand and generic name, usage, regime, effectiveness, and all possible side effects or interactions. |
| **Who is responsible** | - Mainly nurses print and explain the discharge documents - Sometimes the explanations are handled by doctors or pharmacists if necessary | - Mainly nurses print and explain the discharge slip - Doctors or nurses are responsible for printing and explaining PDIS forms - Sometimes the explanations are handled by pharmacists if necessary |
| **What’s the content** | - Mainly focus on medication usage/ the changes compared to the admission, and follow-up appointments. - No standardized information on medications’ side effects. - Nurses sometimes provide handwritten notes/ leaflets to discharged patients/ caregivers for self-care management. | - Advise to use PDIS as a reference to communicate with discharged patients/ caregivers for explaining the medications, side effects/ warning signals, and appointments. - Nurses sometimes provide leaflets to discharged patients/ caregivers for self-care management. |
| **What’s the process** | - Mandatory provision of discharge summary (English version only) and verbally explain the content - Voluntarily provide handwritten notes in Chinese for remarks on important issues to discharged patients/caregivers, especially the medications. | - Mandatory provision of discharge summary (in English) together with PDIS (in Chinese) form for discharged patients/ caregivers. - Mandatory provision of verbal explanations to all the contents to the discharged patients or caregivers. - No teach-back is required |
