# Supplementary material for: A Comparison of Patient and Provider Perspectives on an Electronic Health Record–Based Discharge Communication Tool: Survey Study
Source: JMIR Aging. 2025 Jan 29;8:e60506. doi: 10.2196/60506 (PMC11796482; doi:10.2196/60506)
Supplement: Multimedia Appendix 2 [file aging-v8-e60506-s002.docx]

**Additional file 3. Indirect effects for the model**

| **Relationship** | **Standardized estimates** | **95% CI** | **Relative mediation effect** |
| --- | --- | --- | --- |
| Design quality 🡪 Satisfaction | 0.544 | 0.482-0.605 | 30.8% |
| Design quality 🡪 Behavior intention | 0.770 | 0.700-0.841 | 15.1% |
| Perceived usefulness 🡪 Behavior intention | 0.228 | 0.185-0.272 | 32.5% |
