# Supplementary material for: A Comparison of Patient and Provider Perspectives on an Electronic Health Record–Based Discharge Communication Tool: Survey Study
Source: JMIR Aging. 2025 Jan 29;8:e60506. doi: 10.2196/60506 (PMC11796482; doi:10.2196/60506)
Supplement: Multimedia Appendix 3 [file aging-v8-e60506-s003.docx]

**Additional file 4. Total effects for the model**

| **Relationship** | **Standardized estimates** | **95% CI** |
| --- | --- | --- |
| Design quality 🡪 Perceived usefulness | 0.955 | 0.923-0.987 |
| Design quality 🡪 Satisfaction | 0.786 | 0.748-0.824 |
| Perceived usefulness 🡪 Satisfaction | 0.569 | 0.487-0.651 |
| Design quality 🡪 Behavior intention | 0.907 | 0.862-0.952 |
| Perceived usefulness 🡪 Behavior intention | 0.705 | 0.633-0.776 |
| Satisfaction 🡪 Behavior intention | 0.402 | 0.288-0.515 |
| Behavior intention 🡪 Actual behavior | 0.164 | 0.131-0.197 |
